# Social support and mental health among adolescents in Kenya, Indonesia, and Vietnam: A latent class analysis using the National Adolescent Mental Health Surveys

**DOI:** 10.1186/s13034-025-00923-3

**Published:** 2025-07-31

**Authors:** Joemer C. Maravilla, Shoshanna L. Fine, Astha Ramaiya, Mengmeng Li, Yohannes Dibaba Wado, Amirah Ellyza Wahdi, Sally Atieno Odunga, Vinh Duc Nguyen, Harvey A. Whiteford, David Lawrence, James G. Scott, Holly E. Erskine

**Affiliations:** 1 School of Public Health, The University of Queensland, Herston, QLD Australia; 2 Queensland Centre for Mental Health Research, Wacol, QLD Australia; 3 Institute of Health Sciences and Nursing, Far Eastern University, Manila, Philippines; 4 Department of Mental Health, Johns Hopkins Bloomberg School of Public Health, Johns Hopkins University, Baltimore, MD USA; 5 Department of Population, Family and Reproductive Health, Johns Hopkins Bloomberg School of Public Health, Johns Hopkins University, Baltimore, MD USA; 6 African Population and Health Research Center, Nairobi, Kenya; 7 Center for Reproductive Health, Faculty of Medicine, Public Health, and Nursing, Universitas Gadjah Mada, Yogyakarta, Indonesia; 8 Department of Biostatistics, Epidemiology, and Population Health, Faculty of Medicine, Public Health, and Nursing, Universitas Gadjah Mada, Yogyakarta, Indonesia; 9 Institute of Sociology, Vietnam Academy of Social Sciences, Hanoi, Vietnam; 10 Institute for Health Metrics and Evaluation, University of Washington, Seattle, WA USA; 11 School of Population Health, Curtin University, Perth, WA Australia; 12 Child Health Research Centre, The University of Queensland, South Brisbane, QLD Australia; 13 Child and Youth Mental Health Service, Children’s Health Queensland, South Brisbane, QLD Australia

**Keywords:** Social support, Social network, Adolescents, Primary caregiver, Parents, Peers, Mental health, Suicidal ideation, Self-harm, Kenya, Indonesia, Vietnam, Latent class analysis

## Abstract

**Background:**

There is a lack of country-level evidence for the association between social support and adolescent mental health while existing studies vary greatly in how they account for the interplay of multiple sources of social support.

**Methods:**

This study utilised data from the National Adolescent Mental Health Surveys, nationally representative surveys of adolescents aged 10–17 years and their primary caregiver in Kenya, Indonesia, and Vietnam. Patterns of social support among adolescents in each country were assessed using a latent class analysis. The association between the identified social support classes and any mental disorder, suicidal ideation, and self-harm in the past 12 months was assessed using the Bolck–Croon–Hagenaars method, adjusted for demographic characteristics and caregiver mental health. All estimates were weighted to the respective country’s population and presented with 95% confidence intervals (CIs).

**Results:**

Three latent social support classes were consistently identified in Kenya, Indonesia, and Vietnam: *Caregiver-focussed support*, *Other support*, and *Limited support.* The *Caregiver-focussed support* class had the highest proportion of adolescents in all three countries (Kenya: 65.3%, 95% CI: 63.0–67.5; Indonesia: 54.0%, 95% CI: 50.4–57.6; Vietnam: 81.6%, 95% CI: 79.0–84.1), although the proportions varied significantly by country. Adolescents in the Caregiver-focussed support class had significantly lower odds of any mental disorder (Kenya: adjusted odds ratio [aOR]: 0.31, 95% CI: 0.25–0.38; Indonesia: aOR: 0.23, 95% CI: 0.17–0.31; Vietnam: aOR: 0.39, 95 CI%: 0.26–0.57), suicidal ideation (Kenya: aOR: 0.14, 95% CI: 0.10–0.19; Indonesia: aOR: 0.17, 95% CI: 0.10–0.29; Vietnam: aOR: 0.42, 95% CI: 0.24–0.76) and self-harm (Kenya aOR: 0.07, 95% CI: 0.04–0.13; Indonesia aOR: 0.23, 95% CI: 0.11–0.47 and Vietnam aOR: 0.16, 95% CI: 0.09–0.27) compared to the Limited support class. Adolescents belonging to the Other support class also demonstrated lower odds of these outcomes than those in the Limited support class.

**Conclusions:**

The association between social support and poor mental health indicates the critical role of primary caregivers, other family members, and peers in adolescent mental health. These findings highlight the need to develop interventions that leverage an adolescent’s existing support networks.

**Supplementary Information:**

The online version contains supplementary material available at 10.1186/s13034-025-00923-3.

## Background

Social support is broadly defined as informal emotional support, information or advice, or practical assistance to which individuals perceive they have access within their social networks [1–3]. Adolescents often receive support from multiple interrelated sources such as family, peers, romantic partners, educators, employers, neighbours, or faith-based groups [4–6]. These sources of social support often evolve as adolescents mature into young adults and remodel their social relationships [4, 5]. The presence of social support can significantly influence adolescent mental health, both in the short- and long-term [4, 5, 7]. Positive social support can moderate the effect of stress on psychological wellbeing [4, 8] and may also function to improve emotional regulation, self-esteem, and self-worth [9]. This is particularly true in adolescence, where major transitions to new social roles occur and the risk of mental health problems peaks [10, 11].

The majority of studies investigating the association between social support and mental health in adolescents have been conducted in high-income countries (HICs) [2, 12]. A meta-analysis of studies from 49 HICs found significant association between increased levels of social support from family members (odds ratio [OR]: 0.48, 95% confidence interval [95% CI]: 0.40–0.57) and peers (OR: 0.72; 95% CI: 0.66–0.79) and reduced odds of adverse psychosocial outcomes including internalising behaviour, substance use, and bullying involvement [2]. A school-based cohort study in Norway found that depression and anxiety scores significantly decreased as social support from family and friends increased [13]. Similarly, a community-based cohort study in Canada found that high levels of perceived social support at the age of 19 years were associated with lower odds of suicidal ideation (OR: 0.59; 95% CI: 0.50–0.70) and suicide attempt (OR: 0.60; 95% CI: 0.46–0.79) at the age of 20 years [7].

Available studies in low- and middle-income countries (LMICs) also found significant correlations between family and peer support and adolescent mental health, although far fewer studies are available as compared to HICs. For example, a recent study used data from the Global School-based Health Survey (GSHS) to investigate the association between parental and peer support and suicidal ideation among adolescents from 53 HICs and LMICs [14]. Overall, significantly lower odds of suicidal ideation in the past 12 months were observed among those who reported parental support (OR: 0.80; 95% CI: 0.67–0.95) or peer support (OR: 0.59; 95% CI: 0.50–0.69) compared to those who did not report any social support. Further, the effects of each type of support were accounted by elimination; for example, a weaker association was found among adolescents who endorsed peer support but not parent support.

Similar methodological challenges were observed in most studies from HICs and LMICs. Very few studies were nationally representative, while several have focussed on populations with specific experiences or characteristics, such as in-school youth [14], outpatient populations [7, 15–17], disaster survivors [18], or orphaned adolescents [19]. Other studies primarily considered support sources in isolation [14, 20, 21] which meant that the interaction between multiple sources of social support was not considered. As a result, the findings of these studies do not necessarily reflect how different forms of social support can influence each other and, subsequently, mental health and related outcomes.

The operationalised definition of social support varies greatly between studies, making it challenging to generate broad conclusions about the association between social support and adolescent mental health. For example, several studies count the number of available individuals within an adolescent’s social network or use measures which broadly assess the quality of social relationships [2, 12]. However, these methods are unable to consider the interplay of different sources of social support [22]. Assessing the overlap between different sources of support is essential, particularly in contexts where key social supports may also be found outside of family and friends, such as faith-based support systems [6, 23]. Other studies have used scales to generate scores indicating the strength of social support from different sources [2, 24, 25]. While this approach produces a multi-dimensional measure of social support, it assumes that adolescents are a homogenous group and does not consider how patterns of social support may vary across socio-cultural backgrounds.

A comprehensive approach to profiling and quantifying social support is needed to better assess the association between social support and adolescent mental health. Latent class analysis (LCA) is one such method, whereby a modelling approach is applied to address sample heterogeneity by generating latent subgroups or classes. Instead of using a score threshold, LCA assigns an individual to a subgroup or class using probabilities based on a set of observed social support indicators. This person-centred approach characterises social support through complex combinations of social support parameters improving data utility in cross-country research [26]. By identifying co-occurring and nuanced patterns of support, LCA provides foundational evidence for future research to develop and test theoretical models of social support and adolescent mental health in diverse cultural contexts.

The National Adolescent Mental Health Surveys (NAMHS) collected nationally representative data on adolescent mental health and associated factors in Kenya, Indonesia, and Vietnam [27, 28]. As part of the survey instrument in all three countries, questions related to social support were included, encompassing who the adolescent spoke to when having worries or concerns, their perceived relationship with their primary caregiver, and support from their faith community. This study utilised data from NAMHS to identify patterns of social support available to adolescents in Kenya, Indonesia, and Vietnam using LCA. Further, this study investigated the association between latent social support classes and mental disorders, suicidal ideation, and self-harm in all three countries.

## Methods

### Sample

This study used data from NAMHS, which are nationally representative household surveys of adolescents aged 10–17 years in Kenya, Indonesia, and Vietnam. Using a multi-stage sampling design, households were randomly selected and approached by trained lay interviewers. One adolescent and their primary caregiver who lived in the same household were selected, with the adolescent randomly selected if more than one adolescent in scope resided at the household. Consent and assent were sought from the primary caregiver and adolescent, respectively. The primary caregiver and the adolescent were then interviewed using a tablet or smartphone, separately and privately.

A total of 5,155, 5,664, and 5,996 primary caregiver-adolescent pairs completed the survey in Kenya, Indonesia, and Vietnam, respectively. Detailed information about the NAMHS sampling procedure, the development and adaptation of the survey instrument, and data collection is reported in other publications [27, 28].

### Measures

#### Social support

Social support was assessed by five questions which were asked of the adolescent (Table 1). Three questions asked about the adolescent’s relationship with their primary caregiver. One question asked about perceived support from the adolescent’s faith community while the remaining question asked who the adolescent usually spoke to about worries or concerns. The latter question allowed multiple responses which were collapsed into four categories (i.e., primary caregiver, other family members, peers, and others), giving a total of eight indicators of social support from the five questions. The responses to these indicators were dichotomised to improve the interpretability of the estimates and facilitate further analytical procedures. Weighted proportions and unweighted numbers of adolescents who endorsed each indicator in Kenya, Indonesia, and Vietnam are available in Table S1 in the Supplementary Materials.

#### Mental disorders

Mental disorders were assessed using the Diagnostic Interview Schedule for Children, Version 5 (DISC-5) [29, 30]. The DISC-5 is a structured diagnostic interview administered by lay interviewers, i.e., those without clinical training. The DISC-5 provides a diagnosis of mental disorders according to the Diagnostic and Statistical Manual of Mental Disorders, Fifth Edition (DSM-5) [31]. For NAMHS, six diagnostic modules were included which assessed the prevalence of the given mental disorder in the past 12 months: social phobia, generalised anxiety disorder, major depressive disorder, conduct disorder, posttraumatic stress disorder (PTSD), and attention-deficit/hyperactivity disorder (ADHD). These disorders were chosen based on their known associated burden [28, 32]. All modules were administered to the adolescent except for ADHD, which was administered to the primary caregiver. For the purposes of this paper, adolescents who met diagnostic criteria for at least one of the six disorders in scope were grouped into a single category of any mental disorder in the past 12 months.

#### Suicidal ideation and self-harm

Adolescents were asked about suicidal ideation in the past 12 months: ‘During the past 12 months, have you ever thought about attempting suicide?’ Self-harm in the past 12 months was assessed using this question, ‘During the past 12 months, have you deliberately harmed or injured yourself without intending to end your own life?’ All participants were read a short definition of suicide and of self-harm before answering the relevant questions (shown in Text S1 in the Supplementary Materials).

#### Covariates

All demographic information was reported by the primary caregiver. This included adolescent sex (male and female), adolescent age (grouped as 10–14 years and 15–17 years), adolescent school enrolment status (dichotomised as currently attending school or not), and household wealth (analysed as wealth quintiles) [28]. Urbanicity (urban and rural) was determined by the household’s location [27]. Primary caregiver mental health was assessed using the Patient Health Questionnaire-9 (PHQ-9) (coded as minimal, mild, moderate, moderately severe, and severe) and Generalised Anxiety Disorder-7 (GAD-7) (coded as minimal, mild, moderate, and severe) [27], which measured the primary caregiver’s depressive and anxiety symptoms, respectively.

**Table 1:** Social support questions and indicators

Item	Responses	Binary Indicator
How comfortable do you feel talking with your main caregiver about things that worry you?	a. Very comfortable b. Somewhat comfortable c. Not very comfortable d. Not at all comfortable e. Don’t know f. Prefer not to say	Comfortable talking with *primary caregiver* about their worries. • Yes (a, b) • No (c, d) • *Missing (e*,* f)*
Do you feel that your main caregiver cares about what you are thinking and feeling?	a. A lot b. Somewhat c. Not much d. Not at all e. Don’t know f. Prefer not to say	Felt that *primary caregiver* cares about what they are thinking and feeling. • Yes (a, b) • No (c, d) • *Missing (e*,* f)*
Do you feel close to your main caregiver?	a. A lot b. Somewhat c. Not much d. Not at all e. Don’t know f. Prefer not to say	Felt close to their *primary caregiver*. • Yes (a, b) • No (c, d) • *Missing (e*,* f)*
How much do you agree or disagree with the following statement: I feel connected to and supported by my faith community.	a. Strongly agree b. Agree c. Neither agree nor disagree d. Disagree e. Strongly disagree f. I don’t belong to a faith community g. Don’t know h. Prefer not to say	Felt connected to *faith community*. • Yes (a, b) • No (c, d, e, f) • *Missing (g*,* h)*
Who do you usually talk to if you have worries or concerns? *(Multiple responses allowed)*	Primary caregiver	Usually talked to *primary caregiver* about their worries • Yes • No
	Other family member, not primary caregiver • Mother* • Father* • Sibling* • Grandparent* • Other family member*	Usually talked to *other family members* about their worries • Yes (any from the list) • No (none from the list)
	Peers • Friend • Girlfriend or boyfriend	Usually talked to *peers* about their worries • Yes (any from the list) • No (none from the list)
	Others** • Teacher or other school staff member • Doctor, nurse, or health worker • Religious/faith leader • Other	Usually talked to *others* about their worries • Yes (any from the list) • No (none from the list)

Note: *These were regarded as ‘other family members’ if they were not the primary caregiver. ** This list was collapsed into a single group called ‘Others’ due to small cell size (< 5%)

### Analysis

#### Test of association

To test the association between social support classes and any mental disorder, suicidal ideation, and self-harm in the past 12 months, the Bolck–Croon–Hagenaars (BCH) method with modal assignment was undertaken [36]. This modelling technique has been found to produce less biased estimates when testing the relationship between latent classes and an observed distal outcome [37].

The estimated outcome probabilities for each social support class were considered as weighted prevalence in this paper. Adjusted ORs (aORs) and 95% CIs for each outcome were estimated using conditional logit probabilities [36]. All models were weighted and adjusted for demographic characteristics and primary caregiver mental health.

All analyses were conducted using Stata17.0 [38], and the LCA Stata Plug-in v. 1.2.1 [39] with the LCA_Distal Stata function [40].

#### Identification of latent classes of social support

The eight indicators of social support were analysed using LCA. A series of models with an increasing number of classes, from two-class to six-class models, were tested within each country. Maximum likelihood and expectation-maximization algorithms were used to generate latent classes. These parameters include latent class membership posterior probabilities (i.e., latent class proportions) and item-response probabilities (i.e., probabilities of reporting an indicator conditional on class membership).

A class model (i.e., the number of classes of best fit) was selected using multiple fit statistics, including Bayesian information criterion (BIC), sample-size adjusted Bayesian information criterion (aBIC), Akaike information criterion (AIC), bootstrapped likelihood ratio test (BLRT), and sample class size (i.e., classes should not contain less than 5% of the sample). Entropy was also considered to assess the model accuracy on class enumeration [33]. The interpretability of the classes and the external validity in relation to distal outcomes (i.e., any mental disorder in the past 12 months, suicidal ideation in the past 12 months, and self-harm in the past 12 months) were also assessed. External validation investigated the probabilities of distal outcomes conditional to latent classes identified by class models being evaluated, a common procedure when selecting optimal class solutions [34]. This validation procedure was only undertaken after the latent classes were identified. It was not undertaken iteratively; no parameters in the LCA were altered or omitted based on these results.

Full information maximum likelihood estimation was applied to handle missing indicator data as missing at random [35]. All estimates were weighted using the relevant population weight for each country [28]. Item-response probabilities and class membership probabilities on the selected model solution were adjusted for adolescent’s demographic characteristics and primary caregiver mental health. None of the covariates had missing data except for primary caregiver mental health. Missing data were found as missing completely at random after β parameter test. Less than 9% of adolescents were excluded from the analysis due to missing data in any country (Kenya: 1.4%; Indonesia: 4.0%.; Vietnam: 8.2%).

## Results

### Identification of social support classes

Fit statistics used for model selection are shown in Table 2. In Kenya, only the three-class model met an acceptable entropy of above 0.80 [33]. In Indonesia, BIC, aBIC and entropy supported the three-class model. In Vietnam, the three-class model also had the highest entropy with a slightly higher aBIC compared to other class models. Estimates from BLRT further supported the three- and four-class models in Indonesia but were not informative for model selection in Kenya and Vietnam. Class size was also considered to ensure adequate statistical power for further analysis of the association with mental disorders, suicidal ideation, and self-harm which are relatively uncommon outcomes, particularly in Vietnam [28]. The five- and six-class models in all three countries had a class size of less than 5%.

Based on all fit criteria, the three-class model outperformed other class models for Indonesia. Despite slightly better fit statistics in the four-class solution for Vietnam, the greater class sizes in the three-class model made it the better model solution. For Kenya, both the three-class model and the four-class model were potential solutions. Further investigation of the item-response probabilities of both models revealed two classes in the four-model class lent themselves to a similar interpretation. The examination of the external validity of the four-class solution found no significant differences in the conditional probabilities for any mental disorder, suicidal ideation, and self-harm between these two classes. For these reasons, the three-class model was chosen for Kenya.

**Table 2:** Model fit statistics of latent class models by country

Number of classes	LL	BIC	aBIC	AIC	SCS	Entropy	BLRT
Kenya							
2	-15706.78	1760.30	1706.28	1648.99	30.27%	0.77	0.01
3	-15135.55	694.77	612.15	524.52	11.64%	0.93	0.01
4	-15063.24	627.07	515.85	397.90	11.62%	0.72	0.01
5	-15024.30	626.12	486.31	338.02	3.52%	0.75	0.01
6	-15001.35	657.17	488.75	310.14	3.45%	0.70	0.01
Indonesia							
2	-17256.77	936.98	882.96	824.07	32.37%	0.66	0.01
3	-17007.28	515.78	433.16	343.09	13.99%	0.82	0.01
4	-16984.69	548.38	437.16	315.91	10.66%	0.67	0.18
5	-16968.06	592.88	453.06	300.64	4.91%	0.72	0.01
6	-16950.17	634.89	466.47	282.87	1.84%	0.73	0.07
Vietnam							
2	-17475.55	1730.82	1676.80	1616.94	25.95%	0.71	0.01
3	-16904.78	667.57	584.95	493.40	8.34%	0.92	0.01
4	-16862.66	661.61	550.39	427.15	6.71%	0.90	0.01
5	-16831.28	677.14	537.32	382.39	0.78%	0.89	0.01
6	-16805.08	703.04	534.62	348.00	0.76%	0.89	0.01

Note: LL = Log-likelihood, BIC = Bayesian information criterion; aBIC = sample-size adjusted BIC; AIC = Akaike information criterion; SCS = Smallest class size (%); BLRT = p-value from bootstrapped likelihood ratio test

**Fig. 1:**
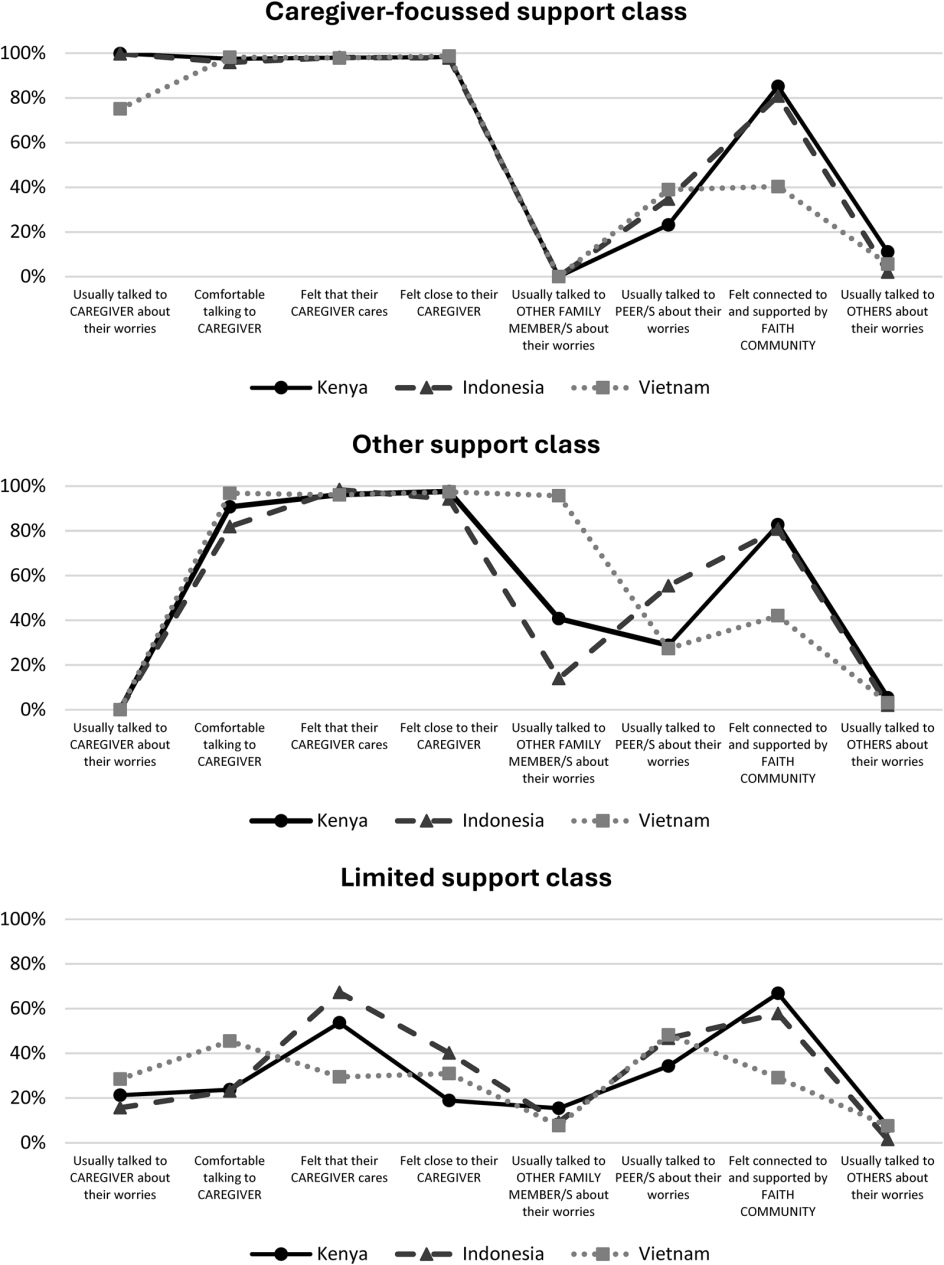
Item-response probabilities for Kenya, Indonesia, and Vietnam by class in the three-class model

Three social support classes generated for Kenya, Indonesia, and Vietnam using LCA. These were labelled the *Caregiver-focussed support* class, *Other support* class, and *Limited support* class. The characteristics of each class are shown in Fig. 1 (with Figure S1 in the Supplementary Materials providing an alternate configuration i.e., the three classes within country).

Due to the skewed number of caregiver support items, a sensitivity analysis with balanced items was undertaken where the three adolescent-caregiver relationship questions (i.e., the first three indicators in Table 1) were omitted. This analysis supported the choice of the three-class model in Kenya and Indonesia (shown in Table S2 in the Supplementary Materials). The reduction of LCA parameters in Vietnam changed the class solution to four classes. External validation of the *Caregiver-focussed support* class found no significant differences in the conditional probabilities for all outcomes between the four-class LCA with reduced parameters and the three-class LCA with the original set of parameters (shown in Table S3 in the Supplementary Materials); hence, the latent classes from three-class solution was retained.

The majority of adolescents in the *Caregiver-focussed support* class showed high likelihood of accessing support from their primary caregiver (Kenya: 99.9%, Indonesia: 99.8%, Vietnam: 75.1%) and endorsed at least one indicator of a strong caregiver-adolescent relationship (Kenya: 97.5 − 98.2%, Indonesia: 95.9 − 98.2%, Vietnam: 97.9 − 98.7%). Compared to other classes, adolescents in *Caregiver-focussed support* class did not tend to seek support from other family members, with this evident across all three countries. In Indonesia and Kenya, adolescents in *Caregiver-focussed support* class had the lowest probability of seeking support from peers (34.8%) and other sources (e.g., teachers, health professionals, and faith leaders) (11.2%), respectively (see Fig. 1).

Across all three countries, adolescents in the *Other support* class showed high likelihood of seeking support from other family members, peers, their faith community, and others. Adolescents in this class did not tend to endorse discussing their worries or concerns with their primary caregiver (<0.1% in all countries) despite indicating a relatively strong caregiver-adolescent relationship (Kenya: 90.7 − 97.6%; Indonesia: 82.0 − 98.4%; Vietnam: 96.1 − 97.4%). The *Other support* class differed between countries on some specific attributes. In Indonesia, endorsement of peer support in this class was more evident (55.4%) than in the *Caregiver-focussed support* class (37.8%). In Kenya and Vietnam, the defining difference between the *Caregiver-focussed support class* and the *Other support* class was a greater tendency to seek support from other family members in the latter (Kenya: 40.8% vs. <0.01%; Vietnam: 95.7% vs. <0.1%). The proportion of adolescents who felt connected to and supported by their faith community was similar between the *Caregiver-focussed support* class (Kenya: 85.2%, Indonesia: 80.8%, Vietnam: 40.4%) and the *Other support* class (Kenya: 82.7%, Indonesia: 80.8%, Vietnam: 42.1%) in each country.

Adolescents in the *Limited support* class generally reported a weak caregiver-adolescent relationship and demonstrated the lowest item-response probabilities for indicators of primary caregiver and faith community support. When compared to the *Other support* class, the *Limited support* class demonstrated lower support from other family members across countries (Kenya: 15.4% vs. 40.8%; Indonesia: 9.2% vs. 13.9%; Vietnam: 7.8% vs. 95.7%). Probabilities for peer support in the *Limited support* class was slightly higher than the *Other support* class in Kenya (34.3% vs. 28.8%) and Vietnam (48.3% vs. 39.0%) but lower in Indonesia (46.7% vs. 55.4%).

### Social support class membership

**Fig. 2:**
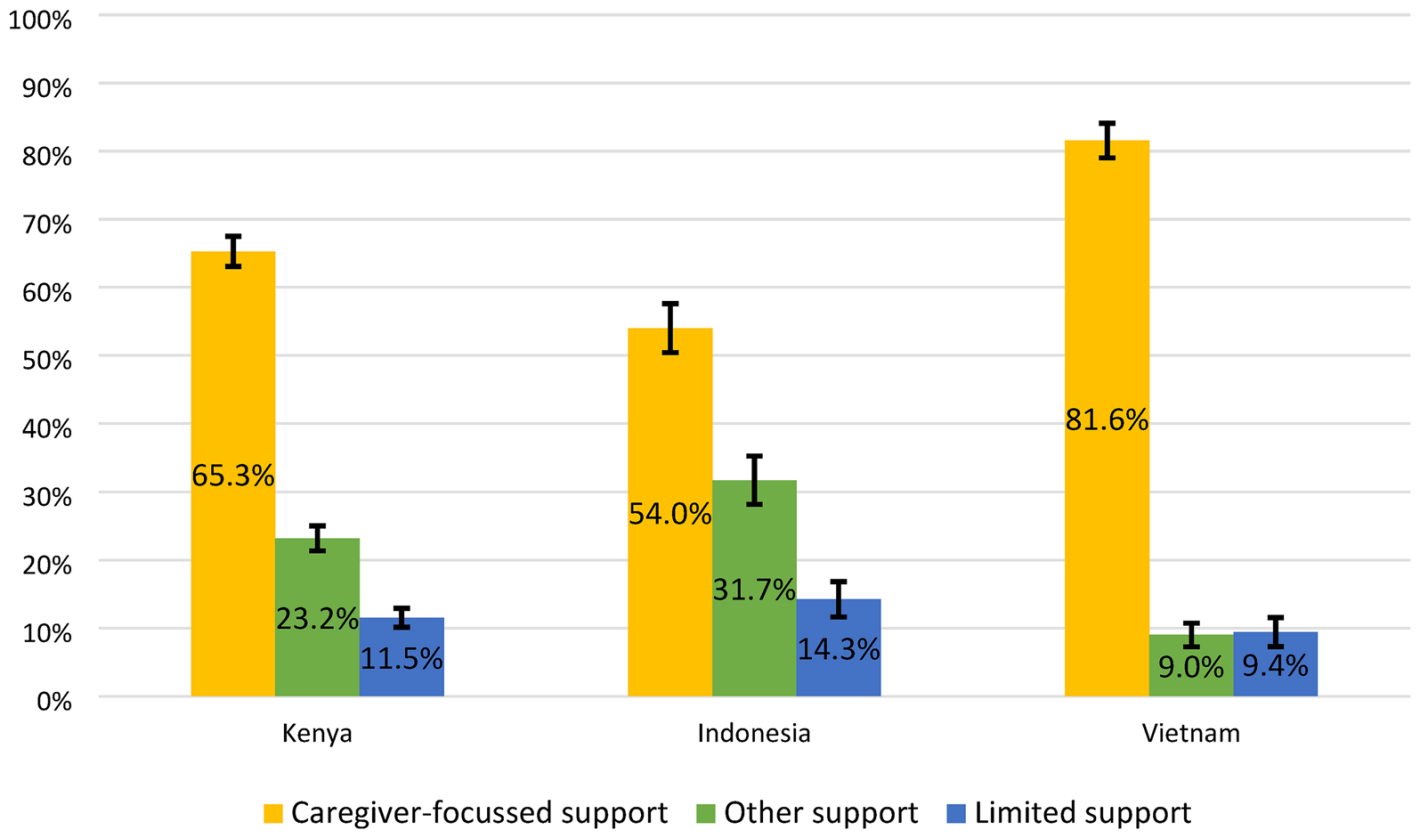
Proportion of adolescents in each social support class in Kenya, Indonesia, and Vietnam

As shown in Fig. 2, the majority of adolescents in all three countries belonged to the *Caregiver-focussed support* class, followed by the *Other support* class, and the *Limited support* class. However, the proportion of adolescents in the *Caregiver-focussed support* class (Vietnam: 81.6%, 95% CI: 79.0–84.1; Kenya: 65.3%, 95% CI: 63.0–67.5; Indonesia: 54.0%, 95% CI: 50.4–57.6) and *Other support* class (Indonesia: 31.7%, 95% CI: 28.2–35.3; Kenya: 23.2%, 95% CI: 21.4–25.0; Vietnam: 9.0%, 95% CI: 7.3–10.7) varied significantly by country. Proportions of adolescents in the Limited support class were relatively consistent across countries (Indonesia: 14.3%, 95% CI: 11.7–16.8; Kenya: 11.5%, 95% CI: 10.1–12.9; Vietnam: 9.4%, 95% CI: 7.3–11.6).

### Association between social support and mental disorders

As shown in Table 3, the prevalence of any mental disorder in the past 12 months was significantly lower among adolescents in the *Caregiver-focussed support* class (Kenya: 9.5%, 95% CI: 8.3–10.7; Indonesia: 3.5%, 95% CI: 2.5–4.4; Vietnam: 2.9%, 95% CI: 2.2–3.6) as compared to those in the *Limited support* class (Kenya: 25.5%, 95% CI: 21.0–30.0; Indonesia: 13.4%, 95% CI: 9.5–17.2; Vietnam: 7.2%, 95% CI: 4.1–10.3) (see Table 3). Further, the prevalence of any mental disorder in the past 12 months was significantly lower among the *Other support* class as compared to the *Limited support* class in Kenya (13.0%, 95% CI: 10.6–15.4) and Indonesia (5.0%, 95% CI: 3.2–6.8).

The odds of experiencing any mental disorder in the past 12 months was significantly lower for those adolescents in the *Caregiver-focussed support* class (Kenya aOR: 0.31, 95% CI: 0.25–0.38; Indonesia aOR: 0.23, 95% CI: 0.17–0.31; Vietnam aOR: 0.39, 95 CI%: 0.26–0.57) and the *Other support* class (Kenya aOR: 0.44, 95% CI: 0.34–0.56; Indonesia aOR: 0.34, 95% CI: 0.25–0.46; Vietnam aOR: 0.54, 95 CI%: 0.30–0.96) as compared to the *Limited support* class in all three countries (Table 3).

**Table 3:** Weighted prevalence and adjusted odds ratios for any mental disorder in the past 12 months by social support class for adolescents in Kenya, Indonesia, and Vietnam

	Kenya	Indonesia	Vietnam
Prevalence, % (95% CI)			
Caregiver-focussed support	9.5 (8.3–10.7)	3.5 (2.5–4.4)	2.9 (2.2–3.6)
Other support	13.0 (10.6–15.4)	5.0 (3.2–6.8)	4.0 (2.0–6.0)
Limited support	25.5 (21.0–30.0)	13.4 (9.5–17.2)	7.2 (4.1–10.3)
Adjusted odds ratio,^+^ aOR (95% CI)			
Caregiver-focussed support	0.31 (0.25–0.38)	0.23 (0.17–0.31)	0.39 (0.26–0.57)
Other support	0.44 (0.34–0.56)	0.34 (0.25–0.46)	0.54 (0.30–0.96)
Limited support	*Ref.*	*Ref.*	*Ref.*

Note: % = weighted prevalence; 95% CI = 95% confidence interval; aOR = adjusted odds ratio (weighted)

^+^Adjusted for adolescent age, adolescent sex, urbanicity, household wealth, adolescent current school attendance, and primary caregiver mental health

### Association between social support and suicidal ideation

In Kenya and Indonesia, the prevalence of suicidal ideation in the past 12 months was significantly lower in the *Caregiver-focussed support* class (Kenya: 2.5%, 95% CI: 2.0–3.1; Indonesia: 0.8%, 95% CI: 0.3–1.3) as compared to the *Limited support* class (Kenya: 15.3%, 95% CI: 11.7–18.9; Indonesia: 4.7%, 95% CI: 2.4–6.9) (Table 4). As shown in Table 4, Kenya and Indonesia demonstrated a difference between the *Other support* class (Kenya: 4.7%, 95% CI: 3.2–6.3; Indonesia: 1.0%, 95% CI: 0.1–1.9) and the *Limited support* class. No significant differences were seen between any classes in Vietnam.

Adolescents belonging to *Caregiver-focussed support* had lower odds of suicidal ideation compared to those in the *Limited support* class (Kenya aOR: 0.14, 95% CI: 0.10–0.19; Indonesia aOR: 0.17, 95% CI: 0.10–0.29; Vietnam aOR: 0.42, 95% CI: 0.24–0.76) after adjustment. Likewise, adolescents in the *Other support* class also had lower odds of suicidal ideation than those in the *Limited support* class (Kenya aOR: 0.27, 95% CI: 0.19–0.39; Indonesia aOR: 0.20, 95% CI: 0.11–0.37; Vietnam aOR: 0.21; 95% CI: 0.06–0.72).

**Table 4:** Weighted prevalence and adjusted odds ratios of suicidal ideation in the past 12 months by social support class for adolescents in Kenya, Indonesia, and Vietnam

	Kenya	Indonesia	Vietnam
Prevalence, % (95% CI)			
Caregiver-focussed support	2.5 (2.0–3.1)	0.8 (0.3–1.3)	1.3 (0.9–1.8)
Other support	4.7 (3.2–6.3)	1.0 (0.1–1.9)	0.7 (< 0.1–1.4)
Limited support	15.3 (11.7–18.9)	4.7 (2.4–6.9)	3.1 (1.0–5.1)
Adjusted odds ratio^+^, aOR (95% CI)			
Caregiver-focussed support	0.14 (0.10–0.19)	0.17 (0.10–0.29)	0.42 (0.24–0.76)
Other support	0.27 (0.19–0.39)	0.20 (0.11–0.37)	0.21 (0.06–0.72)
Limited support	*Ref.*	*Ref.*	*Ref.*

Note: % = weighted prevalence; 95% CI = 95% confidence interval; aOR = adjusted odds ratio (weighted)

^+^Adjusted for adolescent age, adolescent sex, urbanicity, household wealth, adolescent current school attendance, and primary caregiver mental health

### Association between social support and self-harm

In Kenya and Vietnam, adolescents in the *Caregiver-focussed* (Kenya: 0.4%, 95% CI: 0.1–0.7; Vietnam: 0.8%, 95% CI: 0.4–1.2) and *Other support* classes (Kenya: 1.2%, 95% CI: 0.5–2.0; Vietnam: 1.0%, 95% CI: <0.1–2.0) demonstrated significantly lower prevalence of self-harm as compared to the *Limited support* class (Kenya: 5.4%, 95% CI: 3.0–7.7; Vietnam: 4.9%, 95% CI: 2.2–7.5) (Table 5). No significant differences in the prevalence of self-harm by class were seen in Indonesia.

As shown in Table 5, adolescents in the *Caregiver-focussed support* class were significantly less likely to report self-harm in the past 12 months in Kenya (aOR: 0.07, 95% CI: 0.04–0.13), Indonesia (aOR: 0.23, 95% CI: 0.11–0.47) and Vietnam (aOR: 0.16, 95% CI: 0.09–0.27) as compared to in the *Limited support* class. In all three countries, adolescents belonging to the *Other support* class also demonstrated significantly lower odds of self-harm (Kenya aOR: 0.22, 95% CI: 0.12–0.41; Indonesia aOR: 0.38, 95% CI: 0.19–0.79; Vietnam aOR: 0.19, 95% CI: 0.07–0.53) as compared to those in the *Limited support* class.

**Table 5:** Weighted prevalence and adjusted odds ratios of self-harm in the past 12 months by social support class among adolescents in Kenya, Indonesia, and Vietnam

	Kenya	Indonesia	Vietnam
Prevalence, % (95% CI)			
Caregiver-focussed support	0.4 (0.1–0.7)	0.5 (0.2–0.9)	0.8 (0.4–1.2)
Other support	1.2 (0.5–2.0)	0.7 (0.1–1.6)	1.0 (< 0.1–2.0)
Limited support	5.4 (3.0–7.7)	2.2 (0.5–3.9)	4.9 (2.2–7.5)
Adjusted odds ratio^+^, aOR (95% CI)			
Caregiver-focussed support	0.07 (0.04–0.13)	0.23 (0.11–0.47)	0.16 (0.09–0.27)
Other support	0.22 (0.12–0.41)	0.38 (0.19–0.79)	0.19 (0.07–0.53)
Limited support	*Ref.*	*Ref.*	*Ref.*

Note: % = weighted prevalence; 95% CI = 95% confidence interval; aOR = adjusted odds ratio (weighted)

^+^Adjusted for adolescent age, adolescent sex, urbanicity, household wealth, adolescent current school attendance, and primary caregiver mental health

## Discussion

This study is the first to characterise adolescent social support in Kenya, Indonesia, and Vietnam using both nationally representative data and an analytical approach able to account for the distinct patterns and interplay of sources of social support. The LCA revealed three distinct social support classes which were present across all three countries and further demonstrated the same relative ranking within country: *Caregiver-focussed support*,* Other support*,* and Limited support*. In all three countries, adolescents belonging to the *Caregiver-focussed support* and *Other support* classes had significantly lower odds of any mental disorder (aORs: 0.23–0.54), suicidal ideation (aORs: 0.14–0.42), and self-harm (aORs: 0.07–0.38) in the past 12 months as compared to those in the *Limited support* class.

The association between social support and mental health in the current study are in line with those of other studies utilising similar methodological approaches. For example, the US National Longitudinal Study of Adolescent to Adult Health used LCA to investigate the association between social support classes and depressive symptoms [22]. The study found that adolescents who endorsed social support from parents, teachers, and peers had a significantly lower risk of depressive symptoms compared to those with relatively lower support from all sources [22]. However, limited comparative data is available from LMICs. A survey in five public and private high schools in Kenya investigated the independent effects of social support using the Multidimensional Scale of Perceived Social Support Scale [41]. This study reported a significant (unadjusted) correlation between support from family and friends, and depressive and anxiety symptoms. A school-based study in Vietnam sampled adolescents with at least one parent left home for work from rural areas with high number of migrant workers, and found reduced odds for conduct problems among adolescents with high level of support from family (OR: 0.56, 95% CI: 0.43–0.73) and friends (OR: 0.68, 95% CI: 0.52–0.89) [42]. Despite such studies generally showing associations between social support and poor mental health, the use of small-scale samples, focus on specific or special populations, and only including symptom level measures of mental health, limit the external validity of these findings to the general population and their ability to inform public health initiatives [12, 41, 42].

The empirical approach of this study provided a nuanced understanding of adolescents’ social support networks. Instead of describing social support through summative scores and multiple-way interactions which are often challenging to interpret, the current study dissected distinct support networks within and between countries as latent classes. These latent classes provided an initial basis for developing theoretical models to promote adolescent mental health through strategies focussed on strengthening key aspects of social support and engaging families and communities.

The high proportion of adolescents belonging to the *Caregiver-focussed support* class in each country (Kenya: 65.3%; Indonesia: 54.0%; Vietnam: 81.6%). Adolescents from this class were those who reported having a good relationship with their parents and caregivers and approached them for emotional problems. The strong inverse associations between caregiver-focussed support and mental disorders, suicidal ideation, and self-harm indicate the need to engage parents and caregivers in adolescent mental health interventions and public health strategies. However, parents and caregivers remain an untapped sector in low-resource contexts despite its known cost-effectiveness in prevention and early intervention space in high-income countries such as Australia [43]. Active engagement of primary caregivers may provide frontline support within a familial setting. Further, enhancing the parenting skills of primary caregivers may encourage adolescents to seek support from their caregivers [44–46], reducing the risk of mental disorders. In parallel, programs to equip primary caregivers with the ability to recognise signs of poor mental health in their adolescents and the confidence to respond to their adolescents’ concerns or worries may further reduce the risk of mental disorders through upskilling those already in an adolescent’s social support network.

The findings of the current study also indicate that other sources of social support, as represented by the *Other support* class (Kenya: 23.2%; Indonesia: 31.7%; Vietnam: 9.0%), are still important to consider when looking to improve adolescent mental health. This is particularly the case for adolescents who may prefer to disclose their worries and concerns to their grandparents, siblings, or peers, rather than their primary caregiver. In parallel, evidence has shown that primary caregivers can play a significant role in encouraging their children to engage in meaningful friendships with peers and with others in the broader community [14, 47]. This aligns with the characteristics of the *Other support* class as found in this study i.e., a tendency to discuss worries and concerns with others while reporting a positive relationship with their primary caregiver. The results also highlighted the influence of other family members in youth social and emotional development specifically in Vietnam where living with extended family in the same household is common [48].

The findings of the current study further underscores the importance of an integrated approach when identifying adolescent social support networks. For example, in both the l*Caregiver-focussed support* and the *Other support* classes, support from a faith community was a key feature, particularly in Kenya and Indonesia, where faith communities have an important role in parenting and child development [49, 50]. This finding is supported by the existing, albeit limited, literature looking at the role of faith communities in this context. For example, a qualitative study of adolescents recruited from diverse settings in Indonesia revealed parental support, trusting peer relationships, and connectedness to a faith community as key determinants of positive mental health [51]. The potential influence and role of faith communities in providing social support for adolescents [47, 51] could therefore be leveraged to support the mental health of adolescents in their communities and potentially to even disseminate key health messages on adolescent mental health and wellbeing. This research underscores the importance of leveraging informal sources to enable community-based care, particularly in areas where formal services are unavailable and stigma is high [52].

Although the current study represents an important contribution to the understanding of social support and mental health among adolescents in LMICs, there are some limitations that must be considered when interpreting these findings. First, the indicators included in the LCA did not assess the quality of the adolescent’s peer relationship. In light of the evidence for the influence of peers relative to families during adolescence [53], such information may have altered the social support classes defined in this LCA or impacted the association with the reported outcomes. However, it is unlikely that this would have had a significant impact on the *Caregiver-focussed support* class, to which most adolescents belonged and demonstrated the strongest positive associations with mental disorders, suicidal ideation, and self-harm. This was confirmed by a sensitivity analysis with balanced LCA parameters as well as an external validation of the *Caregiver-focussed support* class in model solutions with the reduced and the original set of parameters. Nevertheless, the generalisability of the social support classes identified by this study should be interpreted cautiously, particularly regarding their acceptability and relevance in other sociocultural contexts.

Second, the reported association between the social support classes and mental disorders, suicidal ideation, and self-harm are based on cross-sectional data. Given that previous studies have reported bidirectional relationships between social support and mental health (e.g., those with existing mental health problems may be unable to establish social support networks) [54, 55], the associations reported in this study should be carefully interpreted for an indication of causality. Moreover, this study measured adolescent perception of social support, which is at risk for reporting bias, particularly among those with mental disorders [56]. However, previous longitudinal studies have found social support as a strong predictor of depressive and anxiety symptoms and suicidal behaviours in adolescence after addressing the issue of reverse causality [7, 22], which supports the associations found in this current study. Third, NAMHS did not directly assess online social networks or other online groups as sources of social support. While questions did not specify whether the measured supports were in-person or online, it is possible that the included response options did not represent some online sources of social support. As a result, it is possible that profiles of social support and the association with the reported outcomes may have differed had these online sources been directly measured. Further, this omission also means that information about the relative importance or influence of online social support on adolescent mental health is not available to policymakers. Finally, the current study did not account for the confounding effects of risk factors such as bullying, substance use, and adverse childhood experiences due to these potentially being in the causal pathway. Further, there was insufficient evidence to confirm that including these risk factors in the model would not lead to an over-adjustment.

## Conclusions

The current study found that social support was associated with significantly reduced odds of mental disorders, suicidal ideation, and self-harm among adolescents in Kenya, Indonesia, and Vietnam. The findings of this study identify collective support, particularly from primary caregivers, other family members, peers, and faith communities, as a potential key component of interventions designed to maintain positive adolescent mental health. Research on social support as a protective factor provides opportunities for interventions to leverage existing social networks and further promote mental health [57]. This aligns with the current global call [57–60] to focus on adolescent strengths and positive development by investigating social attributes and developing interventions that foster interpersonal relationships.

## Electronic supplementary material

Below is the link to the electronic supplementary material.

Supplementary Material 1.

## Data Availability

The NAMHS datasets are co-owned by the University of Queensland and each respective in-country lead organisation (K-NAMHS: APHRC and UQ; I-NAMHS: UGM and UQ; V-NAMHS: IOS and UQ). Currently, these datasets or analysis of these datasets are available for collaborative work on request to the relevant data owners following an established protocol. Work is currently underway to convert the NAMHS datasets into public-use datasets, allowing for wide use of these datasets while ensuring protection of participant privacy, adherence to country-specific legislation, and appropriate use of data. This includes development of accompanying meta-data, inclusive of a codebook, technical manual, and analysis files. The expected launch date for these public use datasets and accompanying meta-data is 2026, with hosting mechanisms currently under development in line with country legislation and ethical requirements.

## Abbreviations

ADHD: Attention-deficit/hyperactivity disorder
AIC: Akaike information criterion
aBIC: Adjusted Bayesian information criterion
aOR: Adjusted odds ratio
BIC: Bayesian information criterion
BCH: Bolck–Croon–Hagenaars
CIs: Confidence intervals
DISC-5: Diagnostic Interview Schedule for Children, Version 5
DSM-5: Diagnostic and Statistical Manual of Mental Disorders, Fifth Edition
GAD-7: Generalised Anxiety Disorder-7
GSHS: Global School-based Student Health Survey
HICs: High-income countries
LMICs: Low- and middle-income countries
NAMHS: National Adolescent Mental Health Surveys
OR: Odds ratio
PHQ-9: Patient Health Questionnaire-9
PTSD: Post traumatic stress disorder
