# Supplementary material for: Social support and mental health among adolescents in Kenya, Indonesia, and Vietnam: A latent class analysis using the National Adolescent Mental Health Surveys
Source: Child Adolesc Psychiatry Ment Health. 2025 Jul 31;19(Suppl 1):85. doi: 10.1186/s13034-025-00923-3 (PMC12312257; doi:10.1186/s13034-025-00923-3)
Supplement: Supplementary file 1 — Supplementary Material 1. [file 13034_2025_923_MOESM1_ESM.docx]

**Supplementary Materials**

[**Text S1. Definition of suicide and of self-harm used in the instrument.** 2](#_Toc199792075)

[**Figure S1. Item-response probabilities for the three-class model by country.** 3](#_Toc199792076)

[**Table S1. Weighted prevalence of social support indicators in Kenya, Indonesia, and Vietnam.** 4](#_Toc199792077)

[**Table S2. Model fit statistics of latent class models after omitting adolescent-caregiver relationship parameters by country** 5](#_Toc199792078)

[**Table S3. Conditional probabilities and adjusted odds ratios of Caregiver-focussed support class using the three-class model with the original set of parameters and the four-class model with reduced parameters in Vietnam** 6](#_Toc199792079)

# **Table S1. Weighted prevalence of social support indicators in Kenya, Indonesia, and Vietnam.**

|  | **Kenya** | | **Indonesia** | | **Vietnam** | |
| --- | --- | --- | --- | --- | --- | --- |
|  | **% (95% CI)** | **n** | **% (95% CI)** | **n** | **% (95% CI)** | **n** |
| Usually talked to **primary** **caregiver** about their worries | 67.5  (65.1 - 69.8) | 3461 | 56.1  (52.0 - 60.2) | 3064 | 65.0  (61.1 - 68.8) | 4038 |
| Comfortable talking to **primary** **caregiver** | 87.3  (85.9 - 88.7) | 4474 | 81.2  (78.2 - 83.9) | 4373 | 93.6  (91.6 - 95.2) | 5364 |
| Felt that their **primary** **caregiver** cares | 92.4  (91.3 - 93.4) | 4726 | 93.8  (92.4 - 95.0) | 5048 | 92.1  (90.1 - 93.8) | 5251 |
| Felt close to their **primary** **caregiver.** | 88.9  (87.4 - 90.2) | 4557 | 88.4  (85.8 - 90.6) | 4875 | 92.6  (90.7 - 94.2) | 5326 |
| Usually talked to **other family member/s** about their worries | 11.4  (10.2 - 12.7) | 581 | 5.8  (4.6 - 7.3) | 322 | 8.8  (7.7 - 10.1) | 545 |
| Usually talked to **peer/s** about their worries | 25.8  (23.7 – 28.0) | 1298 | 42.8  (39.4 - 46.4) | 2343 | 38.7  (35.3 - 42.2) | 2457 |
| Felt connected to and supported by **faith community** | 82.4  (81.4 - 84.1) | 4221 | 77.5  (73.7 - 81.0) | 3934 | 40.8  (34.6 - 47.4) | 1522 |
| Usually talked to **others** about their worries | 9.4  (8.2 - 10.8) | 477 | 2.0  (1.5 - 2.7) | 96 | 5.4  (4.3 - 6.7) | 349 |

Note: %=weighted prevalence; 95% CI=95% confidence interval; n=unweighted number of adolescents who endorsed social support indicators

# **Text S1. Definition of suicide and of self-harm used in the instrument.**

**Suicide.** I’m now going to ask you some questions about suicide. Suicide or attempting suicide means taking some action to try and end your own life.

**Self-harm.** Sometimes people feel so distressed, depressed, anxious, or hopeless that they may consider hurting or injuring themselves. Deliberate self-harm means deliberately hurting or injuring yourself without trying to end your life.

| **Kenya** |
| --- |
|  |
| **Indonesia** |
|  |
| **Vietnam** |
|  |
|  |

# **Figure S1. Item-response probabilities for the three-class model by country.**

# **Table S2. Model fit statistics of latent class models after omitting adolescent-caregiver relationship parameters by country**

| **Number of classes** | **BIC** | **aBIC** | **AIC** | **SCS** | **Entropy** |
| --- | --- | --- | --- | --- | --- |
| **Kenya** |  |  |  |  |  |
| 2 | 260.30 | 225.34 | 188.27 | 32.36% | 0.98 |
| 3 | 190.47 | 136.45 | 79.16 | 22.51% | 0.69 |
| 4 | 221.81 | 148.72 | 71.21 | 17.23% | 0.52 |
| 5 | 269.32 | 177.16 | 79.43 | 10.00% | 0.55 |
| 6 | 320.28 | 209.06 | 91.11 | 10.03% | 0.44 |
| **Indonesia** |  |  |  |  |  |
| 2 | 163.04 | 128.08 | 89.98 | 43.51% | 0.96 |
| 3 | 165.82 | 111.80 | 52.91 | 18.44% | 0.78 |
| 4 | 211.34 | 138.25 | 58.58 | 12.31% | 0.45 |
| 5 | 262.26 | 170.11 | 69.64 | 8.95% | 0.40 |
| 6 | 313.99 | 202.77 | 81.52 | 8.10% | 0.34 |
| **Vietnam** |  |  |  |  |  |
| 2 | 283.32 | 248.37 | 209.63 | 34.43% | 0.96 |
| 3 | 271.60 | 217.58 | 157.72 | 6.49% | 0.88 |
| 4 | 236.52 | 163.43 | 82.44 | 8.74% | 0.64 |
| 5 | 277.35 | 185.19 | 83.08 | 11.59% | 0.58 |
| 6 | 317.38 | 206.16 | 82.92 | 3.52% | 0.46 |

Note: BIC = Bayesian information criterion; aBIC = sample-size adjusted BIC; AIC = Akaike information criterion; SCS = Smallest class size (%)

# **Table S3. Conditional probabilities and adjusted odds ratios of Caregiver-focussed support class using the three-class model with the original set of parameters and the four-class model with reduced parameters in Vietnam**

|  | **3-class model** | **4-class model** |
| --- | --- | --- |
| **Conditional probabilities,** % (95% CI) |  |  |
| Any mental disorder in the past 12 months | 2.9 (2.2 - 3.6) | 3.3 (1.9 - 4.7) |
| Suicidal ideation in the past 12 months | 1.3 (0.9 - 1.8) | 0.8 (0.2 - 1.4) |
| Self-harm in the past 12 months | 0.8 (0.4 - 1.2) | 0.3 (<0.1 - 0.6) |
| **Adjusted odds ratio,^+^** aOR (95%CI) |  |  |
| Any mental disorder in the past 12 months | 0.39 (0.26 - 0.57) | 0.56 (0.39 - 0.81) |
| Suicidal ideation in the past 12 months | 0.42 (0.24 - 0.76) | 0.34 (0.18 - 0.65) |
| Self-harm in the past 12 months | 0.16 (0.09 - 0.27) | 0.14 (0.06 - 0.36) |

Note: % = weighted probability; 95% CI = 95% confidence interval; aOR = adjusted odds ratio (weighted), limited support class as reference group; ^+^Adjusted for adolescent age, adolescent sex, urbanicity, household wealth, adolescent current school attendance, and primary caregiver mental health.
